# Supplementary material for: Single Cell Transcriptomic and Chromatin Profiles Suggest Layer Vb Is the Only Layer With Shared Excitatory Cell Types in the Medial and Lateral Entorhinal Cortex
Source: Front Neural Circuits. 2022 Jan 26;15:806154. doi: 10.3389/fncir.2021.806154 (PMC8826650; doi:10.3389/fncir.2021.806154)
Supplement: Supplementary file 14 [file Data_Sheet_1.ZIP › Inh_vs_Exc_Motifs/homerResults/motif4.similar.html]

motif4

## Information for motif4

C
A
T
G
G
T
A
C
G
A
C
T
C
G
T
A
C
G
A
T
C
G
A
T
G
A
C
T
G
C
A
T
G
A
C
T
C
T
G
A
A
C
T
G
G
T
A
C
  
Reverse Opposite:  

C
A
T
G
T
G
A
C
G
A
C
T
C
T
G
A
C
G
T
A
C
T
G
A
G
C
T
A
C
G
T
A
G
C
A
T
C
T
G
A
C
A
T
G
G
T
A
C
  

|  |  |
| --- | --- |
| p-value: | 1e-81 |
| log p-value: | -1.875e+02 |
| Information Content per bp: | 1.668 |
| Number of Target Sequences with motif | 1829.0 |
| Percentage of Target Sequences with motif | 14.94% |
| Number of Background Sequences with motif | 3658.2 |
| Percentage of Background Sequences with motif | 9.48% |
| Average Position of motif in Targets | 258.6 +/- 205.5bp |
| Average Position of motif in Background | 296.5 +/- 250.8bp |
| Strand Bias (log2 ratio + to - strand density) | 0.1 |
| Multiplicity (# of sites on avg that occur together) | 1.09 |
| Motif File: | file (matrix) reverse opposite |

### Similar de novo motifs found

|  |  |  |  |  |  |  |  |
| --- | --- | --- | --- | --- | --- | --- | --- |
| Rank | Match Score | Redundant Motif | P-value | log P-value | % of Targets | % of Background | Motif file |
| 1 | 0.956 | T G A C A G C T C T G A G C T A C G T A G C T A C G T A G C A T C T G A A C T G | 1e-58 | -134.507353 | 18.28% | 13.10% | motif file (matrix) |
| 2 | 0.721 | G T A C G A T C T C A G C T A G C T G A T C G A G C T A C G A T C G T A T C A G | 1e-22 | -52.215434 | 16.69% | 13.53% | motif file (matrix) |
